# Post-Translational Control of Sp-Family Transcription Factors

**DOI:** 10.2174/138920208785133244

**Published:** 2008-08

**Authors:** J.S Waby, C.D Bingle, B.M Corfe

**Affiliations:** School of Medicine and Biomedical Sciences, University of Sheffield, Royal Hallamshire Hospital, Sheffield, S10 2JF, UK

**Keywords:** Sp family, Sp1, Sp3 phosphorylation, acetylation, glycosylation, sumolation.

## Abstract

Sp-family transcription factors are widely expressed in human tissues and involved in the regulation of many cellular processes and response to cellular microenvironment. These responses appear to be mediated by alterations in transcription factor affinity for DNA rather than altered protein level. How might such changes be effected? This review will identify the range of known post-translational modifications (PTMs) of Sp-factors and the sometimes conflicting literature about the roles of PTMs in regulating activity. We will speculate on the interaction between cell environment, chromatin microenvironment and the role of PTM in governing functionality of the proteins and the complexes to which they belong.

## INTRODUCTION

The Sp/KLF family is divided into two major subgroups: the Sp-family, which are highly homologous to Sp1 in the zinc finger region; and the KLF family which are more heterogenous and are named after the Drosophila segmentation gene Kruppel which also contains 3 zinc finger motifs [1]. The Sp-family is made up of 8 genes Sp1-8, each located adjacent to a HOX gene cluster and the KLF family contains 15 known members [2]. Specificity protein/Kruppel-like factor (Sp/KLF) family of transcriptions factors are characterised by the presence of 3 highly conserved zinc finger domains which confer DNA-binding ability. Due to this conserved DNA-binding motif, members of the Sp/KLF family share the same DNA recognition sites, namely GC (GGGGCGGGG) and GT (GGTGTGGGGG) boxes. The affinity of Sp/KLF proteins for these sites varies due to small amino acid sequence changes in the recognition domain.

The regulation of transcription by ubiquitously expressed transcription factors of this family is not generally thought to occur through protein turnover, thereby invoking a key role for post-translational modification of Sp proteins in governing transcriptional activity.

### The SP-Family

Sp1, the first identified member of this family [3, 4] was shown to be a transactivator of the simian virus 40 (SV40) early promoter [5, 6]. Since then seven further members of this family have been identified and were numbered Sp2-8, according to their order of discovery. The functional roles of the members of the Sp family have been investigated to variable degrees with the most data having been collected for Sp1 and Sp3.

Both Sp1 and Sp3 have been shown to exhibit ubiquitous expression, whereas Sp4 expression is restricted to brain and developing testis. Sp2 expression has been observed in a number of cell lines, however, no data is available regarding tissue expression levels [7]. Sp7 was identified as a bone specific transcription factor required for osteoblast differentiation and bone formation [8]. The expression patterns of Sp5, Sp6 and Sp8 have yet to be investigated. The first four members of the Sp-family, Sp1-4, are more closely related to each other than to Sp5-8. Sp1-4 contain an N-terminal activation domain and a C-terminal DNA binding domain. Sp3 also contains an inhibitory domain which is thought to mediate suppression of Sp3 transcription activation. Sp3 is inactive or only weakly active and is thought to act as a repressor for Sp1 activated genes by competing for the same binding sites [9]. Sp5-8 proteins are shorter, lacking the N-terminal activation domain of Sp1-4 which may explain their decreased transcription activation potential. 

Little is known regarding the function of Sp5-8. The creation of knock out mice has provided some insight into the possible regulatory roles of Sp1-4. Sp1 null mice show severely retarded embryonic development and die after embryonic day 10 (E10) [10]. This evidence indicates that Sp1 is essential for normal embryonic development. Targeted homologous recombination of the Sp3 gene produced Sp^-/-^ embryos which exhibited late and impaired tooth and bone development. Sp3^-/-^ mice survived gestation, but died of respiratory failure perinatally [11]. These observations suggest that both Sp1 and Sp3 are involved in developmental regulation of gene expression. Both Sp1 and Sp3 show increased expression in a number of cancers suggesting that these transcription factors are switched back on during cancer cell differentiation. Virus particles are also known to ‘hijack’ Sp1/3 during viral replication and Sp1 appears to be a specific target of the SV40 virus. The function of Sp2 is poorly understood, however, it is clearly separate from the function of other Sp proteins as Sp2 preferentially binds GT boxes not GC boxes [7]. Sp2 overexpression has been noted in late stage human prostate tumours and may be oncogenic [12]. 

Sp4 knock out mice show a complex phenotype, different to that observed in Sp1 and Sp3 null mice. Sp4^-/-^ mice develop normally until birth, however, within four weeks two thirds of pups die. The surviving Sp4^-/-^ mice exhibit retarded growth and males do not breed due to an absence of mounting behaviour, which has been shown to be linked to brain abnormalities [13]. 

The Sp-family transcription factors appear to have diverse roles which require further characterisation. This is especially apparent for Sp5-8 proteins which are poorly understood.

## POST-TRANSLATIONAL MODIFICATIONS

Transcriptional control may be exerted via shutdown of Sp transcription factor translation, however such a crude method of transcriptional control would not be immediately effective as proteosomal degradation of proteins is not instantaneous. A more refined method would be to modify reversibly Sp-family proteins in a manner which affects their efficacy. PTMs, provide a means of changing the protein structure to affect transcription without having to degrade or create de novo transcription factor. There is a significant body of evidence for the post translational modification of Sp-family proteins in the form of phosphorylation, acetylation, glycosylation and sumolation. This evidence will be discussed here, however, as Sp1 and Sp3 are the best studied the majority of post translational modification data comes from these two family members. The high homology between Sp proteins, especially Sp1-4, suggests that roles of post translational modifications in the transcriptional control of one Sp member may also be relevant to others Sp proteins.

### Phosphorylation

Phosphorylation appears to play a key role as a molecular ‘on-off’ switch in a plethora of biological processes. There is a body of evidence to suggest a role for phosphorylation in transcription factor regulation. The initial suggestion that Sp-family transcription factors were phosphorylated came in 1990 from the observation that SV40 infection induced phosphorylation of Sp1 [14]. Since then, investigating the role of phosphorylation in Sp-family transcription factors has centred on the founder member.

The consensus of these studies appears to be that phosphorylation of Sp1 increases GC box affinity and facilitates transcriptional activation (see Tables). The precise mechanism for this has yet to be revealed, however, the majority of phosphorylation sites thus identified in Sp1 are located within the DNA binding zinc finger domain, suggesting that phosphorylation produces a conformational change which facilitates DNA-zinc finger interaction. 

Phosphorylation of Sp1 in response to viral infection has been reported in two further studies. Chun *et al.,* reported that HIV-1 tat protein induces DNA-dependent kinase mediated phosphorylation of human Sp1 at Ser131 [15]. A more recent study identified two specific sites in human Sp1 (Ser56 and Ser101) which are hyperphosphorylated in response to HSV-1 viral infection. This hyperphosphorylation requires the presence of a member of the phosphatidylinositol 3 -like kinase family, Ataxia telangiectasia mutated protein (ATM) [16]. There are multiple explanations for the enhanced phosphorylation state of Sp1 following viral infection. Firstly it is possible that phosphorylation may result in an increased DNA binding affinity, which could mediate viral “hijacking” of the transcriptional machinery. This would fit with the observed increase in activation of a HIV-1 luciferase reporter construct following HIV-1 infection [15]. Conversely, enhanced Sp1 phosphorylation and DNA binding may be a protective effect to increase the transcription of ‘cellular defence’ genes in the infected cell. Thirdly, Sp1 increased GC-box binding may act to activate the apoptotic pathway and initiate death of infected cells. In support of this, Sp1 phosphorylation has been shown to activate RasL transcription [17]. However phosphorylation of Sp1 is obviously more complex than a simple on-off switch model as viral infection induced phosphorylation is reported to have either no effect or an activation effect on transcription [15, 16]. 

Evidence regarding the role of phosphatases in Sp1 transcriptional regulation is contradictory, however, the literature concurs that phosphatase proteins 1 and 2(A) are involved. The majority of evidence suggests that dephosphorylation of Sp1 causes a decrease in DNA binding and reduced transcriptional activation, however a number of studies only infer Sp1 dephosphorylation and do not specify the residues which are dephosphorylated [18-24]. Contradictory to the main body of evidence, PP2A inhibition with okadaic acid was shown to increase Sp1 Phosphorylation and HIV promoter transcription with no observable effect on Sp1 DNA binding. PP2A dephosphorylation of Sp1 has also been reported to increase the association of dephosphorylated Sp1 with the chromatin fraction in a crude chromatin preparation [25]. Furthermore the dephosphorylation of Sp1 has been shown to increase Sp1 binding affinity to an inducible AAAT promoter element [18]. 

Although there appear to be exceptions, generally phosphorylation acts to increase DNA binding and transcriptional activation, whilst dephosphorylation has the opposite effect. The discrepancies between results may suggest further levels of transcriptional control, although the possibility cannot be excluded that the observations are experimental artefacts due to the different conditions used for the promoter assays. It is also possible that these contrasting data may reflect differing roles of phosphorylation in different cellular contexts. Further work is required to clarify the role of transcription factor phosphorylation in viral infection and transcriptional control.

### Acetylation

The histone acetyl transferases (HATs) and histone deacetylases (HDACs) were originally named after their ability to introduce or remove acetyl groups (-CH_3_CHO) at lysine residues of histone proteins. It has since become clear that the function of these proteins is not restricted to histones.

Site directed mutagenesis, coupled with an *in vitro *acetylation assay has demonstrated that Sp1 is acetylated at a single lysine, K703 [26]. Sp3 has also been shown to be acetylated using pan-acetyl antibodies [27]. Mutation of a lysine residue in the Sp3 inhibitory domain can dramatically reduce but not abolish Sp3 acetylation, indicating that Sp3 is acetylated at further lysine residues [27]. Our data (Waby, Chirakkal & Corfe unpublished) indicate that the long form of Sp3 is the actylated form and the shortform is not acetylated, possibly suggesting a role for N-terminal acetylation in Sp3 regulation, at least in colorectal cells.

Whilst it is evident that both Sp1 and Sp3 are acetylated *in vivo*, the functional relevance of this is unclear. Treatment of cells with the HDAC inhibitor trichostatin A (TSA) has been shown to increase Sp1 acetylation levels resulting in increased expression of the TGF beta type II receptor [28]. Specific silencing of each of HDAC1, HDAC2 or HDAC3 using siRNA resulted in increased p21 promoter activity and expression in an Sp3-dependent manner, suggesting that increased acetylation caused increased activation of Sp3 controlled genes [29]. In support of a role for acetylation in Sp1/3 transactivation, increased acetylation of Sp1 by the DNA topoisomerase II poison TAS causes increased GC-box dependent transcription in MCF7 cells [30].

However, recent work has cast doubt upon this simplistic ‘more acetylation = more transcription’ model. Expression of a recombinant K703A Sp1, which cannot be acetylated at lysine 703, leads to increased expression of the 12(s)–lipoxygenase gene [26]. Treatment with the HDAC inhibitors has also been shown to attenuate the expression of cycloxygenase 2 (COX-2) and insulin like growth factor binding protein 3 (IGFBP3) [31, 32]. One possible explanation for these apparently contrasting data, may reside in the fact that Sp1 and Sp3 compete for GC-box binding sites, this competition could potentially be swayed by acetylation modifications. Sp3 is normally a poor transcriptional activator, however, recombinant Sp3, expressed in a system which lacks acetyltransferases, was found to act as a transcriptional activator with similar potency to Sp1 [33]. This hypothesis is further supported by the observation that GAL4-Sp3 but not GAL4-Sp1 is able to induce p21 in a TSA-dependant manner, indicating that the acetylation of Sp3 is important [34].

Transcription factor activity can also be modulated by altered affinity to the binding site. Again, as Sp1 and Sp3 compete for GC-boxes, small alterations in binding affinity could result in altered occupancy at the promoter and alter the gene expression according to whether the resident transcription factor is an activator or repressor. Chromatin immunoprecipitation (ChIP) assays have demonstrated a reduction in binding of Sp1 accompanied by an increase in Sp3 binding at the major vault protein (MVP) promoter following treatment with either TSA or butyrate [35]. A similar switch of Sp1 for SP3 has been observed at the promoter for the pro-apoptotic protein BAK following butyrate treatment [36].

In summary, acetylation of Sp1/3 has profound effects upon gene transcription. These effects seem to be exerted through a combination of altered binding affinity and changes in transactivation potential which alter the balance between Sp1 activation and Sp3 repression. 

### Glycosylation

Glycosylation has long been recognised as a PTM of transcription factors associated with regulation of activity [37]. Glycosylation of Sp1 is most widely studied in regulation of glucose-responsive genes stimulated by deprivation or through insulin response pathways. Goldberg *et al. *(2006) reported glycosylation of Sp1 altered transcriptional activity of Sp1 in glomerular mesangial cells at the PAI-1 promoter [38]. Sp1 became glycosylated in high-glucose conditions. This did not appear to alter the binding affinity for the promoter by EMSA but was associated with increased transcriptional activity of PAI-1. Contrastingly, Sp1 was downregulated by by glucose inhibition in HeLa cells. In this cell line and context the glycosylation of Sp1 was shown to be reciprocal with threonine phosphorylation [39]. In addition to regulating transcriptional activity of Sp1 at specific loci, glycosylation has been implicated in the shuttling of Sp1 between nucleus and cytoplasm, a further mechanism of regulation analogous to NFkB. Brasse-Hagnel *et al. *(2003) demonstrated that upregulation of arginosuccinate synthetase by both glutamine and glucosamine was associated with glycosylation of Sp1 in the cytosol and subsequent translocation to the nucleus for binding to the ASS promoter [40]. A similar observation was made by Majumdar *et al. *(2003) who identified cytosolic glycosylation of Sp1 in H-411E rat hepatoma cell lines underwrote translocation to the nucleus and activation of calmodulin transcription in response to insulin (but not glucagon) [41]. In an elegant follow-up study the same group identified sequential and reciprocal glycosylation of Sp1 following insulin treatment of cells. Sp1 was glycosylated in the cytosol, but this appeared to be a transient effect which was subsequently replaced by phosphorylation. The reciprocity of serine/threonine phosphorylation observed by Kang *et al. *(2003) was elucidated by Majumdar as replacement of glycosylation by phosphorylation at the following sites: serine 613, 642,699, 703 and threonine 641 [42].

Glycosylation of Sp1 has also been implicated in the regulation of protein-protein interactions. Roos *et al. *(1997) used a glycosylated or unglycosylated fragment of Sp1 (SpE aa378-495) to show that binding to TAF-110 and Sp1 was blocked by glycosylation in this region [43]. Further conflicting data over the role of glycosylation was provided by Chou *et al.* who showed that arsenic-responsive gene expression is modulated through and Sp1 response. This was associated with altered glycosylation, but glycosylation of Sp1 alone was insufficient to drive expression of an arsenic response gene (hTERT) [44].

Taken together, the data suggest that glycosylation has a potentially important role in the wider regulation of Sp1 through governing its cellular location and potentially regulating its binding to co-factors. The data seem to suggest that beyond this level, glycosylation may be replaced by phosphorylation as the key regulator of transcriptional activity at the promoter level.

### Sumoylation

SUMO (small ubiquitin-like modifier) is a PTM of proteins occurring at lysines within a recognised motif: I/V-K-X-E. SUMO-1 adds 9kDa to proteins following modification; SUMO2 and 3 may polymerise and thereby add more mass. Sumoylation is implicated in the regulation of protein-protein interactions, cellular localisation and has been implicated in regulation of a number of transcription factors as both activator and repressor.

Sumoylation has been observed in both Sp1 and Sp3 [45, 46]. Using MCF-7E cells Spengler *et al. *(2005) showed that Sp3 and its shorter isoforms (M1, M2) were modified by SUMO through a combination of immunoprecipitaion and overexpression analyses. Their predictions suggested that Sp3 has three potential sumoylation sites: K9, K120 and K551. Site directed mutagenesis identified K551 as the affected residue in Sp3. K551R substitutions made in full-length Sp3 led to only a marginal increase in transactivation of the PSA promoter (ibid.). In contrast the same mutation in the M1 isoform led to a markedly enhanced transactivation activity. This finding was consolidated with a similar study examining the roles of Sp3 isoform sumoylation on transactivation of the SRC-1A promoter [47]. 

There are fewer reports of the effect of sumoylation on Sp1 activity. Spengler and Brattain followed up their Sp3 analysis with identification of sumoylation of Sp1 at K16. The sumoylation is also associated with activation but through enhanced proteolytic cleavage of the inhibitory domain at the N-terminus of Sp1 leading to activation [45]. 

Clearly there is potential for several members of the Sp1 family to be regulated *via *sumoylation, but no consistent picture emerges: for Sp3 effects are isoforms-specific and the known sumoylation site of Sp3 is absent from Sp1. In Sp1 sumoylation regulates other post-translational modification (proteolytic cleavage) which in turn is activating.

## SUMMARY & FUTURE WORK

This review has highlighted the need for further research in this area. Research thus far has been restricted to the founder family member Sp1 with some attention paid to Sp3. However, the majority of PTMs identified are restricted to the highly conserved DNA binding domain which suggests that as these residues are conserved within the Sp family, the PTMs identified for Sp1 and Sp3 may also apply to other Sp proteins. The mechanism of action for these PTMs has yet to be discovered, however the observation of a high density of PTMs in the DNA binding domain suggests that PTMs could act to structurally alter the zinc fingers to increase or decrease DNA binding affinity. PTMs which are located within the DNA binding face of the protein may be more difficult to identify and most certainly will be unable to be purified using ChIP. However, a recent study by Tan *et al. *was able to ChIP phosphorylated Sp1 suggesting that in some cases the phosphorylation is not present in the DNA binding face but may facilitate a conformational change which affects binding efficiency [48].

The observed reciprocity between glycosylation and phosphorylation suggests that post-translation control may not be as simple as PTMs acting as switches. It seems likely that combinations of phosphorylation, acetylation, sumoylation cooperate to produce subtle changes in transcriptional activation, possibly acting more like a rheostat than a binary switch. Future work will need to examine the combinations of effects of PTMs on binding. A further, as yet unexplored area is the effect of local chromatin architecture and whether the same combinations of PTMs in the same cell may have distinct effects on activity at different chromosomal loci. Addressing these questions will require state-of-the-art chromatin immunoprecipitation approaches.

## Figures and Tables

**Fig. (1):**
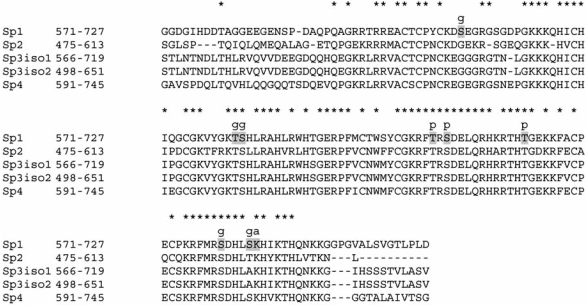
Alignment of Sp-family DNA binding Zinc finger domains. Shaded residue = known post translational modification g= Glycosylation; a=Acetylation sites; p=Phosphorylation sites (nb ser670 and thr668 inferred from rat data).*=conserved residue.

**Table 1.** Phosphorylation of Sp Proteins

Initiation Signal	Cell/Tissue	Associated Kinase/ Phosphatase	Effect on Phosphorylation Including Residues/Location	DNA Binding		Transcription Effects		Ref.
				Probe/Assay	Effect	Promoter	Activation/ Repression	
SV40 infection	HeLa	DNA dependent protein kinase	Phosphorylation of Sp1 N-terminal 610aa (DNA binding domain and transcriptional activation domains)	SV40 promoter	No change	-	-	[14]
HIV-1 Tat protein	HeLa	DNA dependent protein kinase	Ser131	-	-	HIV1-luciferase reporter construct	Activation	[15]
Terminal differentiation	Rat liver tissue	Casein Kinase II	Thr579 and additional sites in the C-terminus aa521-696	Consensus Sp1 sequence	10 fold decreased affinity	-	-	[49]
Cyclin A	NIH3T3 (mouse cell line)	Cyclin A-Cyclin Dependant Kinase 2	Ser59 (corresponds to human Ser61) in N terminal	DHFR promoter fragment	Increased binding	Hamster DHFR	Activation	[50]
Cyclin A	Mouse: U2OS (osteosarcoma); 3T6 (embryonic fibroblast)	CDK2 but CDC2 not excluded	Increased Sp1 phosphorylation at zinc finger domain	Murine TK promoter	Increase both sp1 and sp3	consensus Sp1 site used in a luciferase assay	Activation	[51]
	Human fibrosarcoma and human renal carcinoma cell lines	Atypical protein kinase C, PKC-ζ	Overexpression of PKC- increases SP1 phosphorylation	-	-	VPF/VEGF promoter luciferase reporter construct	Activation2-4 fold increased expression	[52]
CAM induced apoptosis	WKY12-22 and WKY3M-22 (rat aortic smooth muscle cells)	Atypical protein kinase C, PKC-ζ	Phosphorylates Sp1	FasL promoter	Increased phosphorylated Sp1 binding	FasL promoter luciferase reporter construct	Activation	[17]
Angiogenin II	WKY12-22 (rat aortic smooth muscle cells)	Atypical protein kinase C, PKC-ζ	Thr668, Ser670, and Thr681 in zinc finger domain	ChIP p676/686 Sp1	Increased binding at platelet-derived growth factor-D promoter	Platelet-derived growth factor-D	Activation	[48]
P42/p44 MAPK stimulation using estradiol-inducible raf-1 CCL39 cells	CCL39 hamster fibroblast (for EMSA), SL2 Drosophila (for promoter assays)	P42/p44 MAPK	Thr453 (Glutamine rich transactivating domain) and Thr739 (C-terminal D domain) *in vitro* and *in vivo*	Human VEGF promoter	Increased recruitment to promoter	Human VEGF promoter	Activation	[53]
HSV-1 viral infection	Hela; HFF2 (immortalised human foreskin fibroblasts)	Ataxia telangiectasia mutated protein (ATM)	hyperphosphorylates Ser-56 and Ser-101	-	-	CAT assay	No Change	[16]
-	CCRF-CEM a human T-cell leukaemia line and its antifolate resistant sublines	?	Nuclear proteins purified from antifolate resistant cells contained 8 fold more phosphor Sp1	GC box consensus sequence	Dramatic loss of binding	Reduced folate carrier (RFC)	Reduced expression	[54]
Glutaminase antisense RNA	EATC Erlich tumor cells	?	3 fold increase in Sp1 phosphorylation	Sp1 consensus	Inhibition of Sp1–DNA binding	Luciferase reporter construct containing Sp1 consensus ans TATA box	Activation	[55]
Noglamycin treatment	WKY12-22 (rat aortic smooth muscle cells)	PKC-ζ	Induced Sp1 phosphorylation	Sp1/Sp3 consensus sequence	Increased Sp1 binding	Platelet derived growth factor B chain	Activation	[56]
Scleroderma	Human fibroblasts	?	Dermal fibroblasts from patients with Scleroderma show an increased level of Sp1 phosphorylation with no observed difference in overall Sp1 levels this increased phosphorylation is associated with increased expression of the alpha2(I) gene	-	-	-	-	[57]
Okadaic acid stimulation (PP2A inhibitor)	Lymphoblastoid Tcell line	PP2A?	OKA treatment resulted in Sp1 phosphorylation	HIV promoter	No change	HIV promoter	Activation	[58]
T-cell receptor stimulation (TCR)	Human T-cells	PP1 and PP2 INHIBITION by calculin A or okadaic acid	Blockade of PP1 and PP2 increased Sp1 phosphorylation	IL-21R promoter	Decreased	Real time PCR quantification of IL-21R mRNA levels	Reduced TCR-induced IL-21R expression	[59]
Glucose	30A5 (mouse preadipocytes)	PP1	-	Acetyl-CoA carboxylase promoter II	Decrease	Acetyl-CoA carboxylase promoter II	Repression	[19]
Glucose	Hepatoma cells	PP1	-	Aldolase and pyruvate kinase promoters	Decrease	Aldolase and pyruvate kinase promoters	Repression	[20]
Mp1 ligand (thrombopoietin)	Y10/L8057 (megakaryocytic cells)	PP1	-	Cyclin D3	Decrease	Cyclin D3	Repression	[21]
Lysophosphatidylcholine	HUVEC	PP2A	-	Nitric-oxide synthase	Decrease	Sp1 consensus	Repression	[23]
Adipocyte differentiation	3T3-L1 preadipocyte	?	Dephosphorylation of Sp1	Amino acid adipocyte transporter (AAAT) promotor	Increased binding	-	-	[18]
CD2/CD28 costimulation	Human T lymphocytes, Kit225 cells	PP2A	Dephosphorylation of Sp1	HIV-1 LTR 3 Sp motif	Decrease	SV40 early promoter, HIV-1 LTR	Repression	[22]
Lipopolysaccharide (LPS) insult	Mouse lung	?	Dephosphorylation at serine and threonine residues and phosphorylation at a tyrosine residue	Sp1 consensus	Decreased binding	-	-	[24]
Cell cycle interphase	Human cell lines and T cells	PP2A	Dephosphorylation at Ser59, and Thr681	Cell lysis and analysis of chromatin containing fraction	Increased association of dephosphorylated Sp1 with chromatin	-	-	[25]
In vitro treatment of nuclear extracts with dephosphotase	HT29	-	Inferred decreased Sp phosphorylation	AKR1C1 promoter	Decrease	-	-	[60]

**Table 2.** Other Post Translational Modifications of Sp Proteins

Acetylation								
Treatment	Cell/Tissue	Associated HAT/HDAC	Effect on Acetylation Including Residues/Location	DNA Binding		Transcription Effects		Ref.
				Probe/Assay	Effect	Promoter	Activation/Repression	
		P300/ HDAC1	Sp1 K703	-	-	*In vitro* BCAT-2 reporter transcription assay (using Hela nuclear extract)-	Activation-	[27]
Trichostatin A (TSA) treatment-	MCF-7L breast cancer cell lineHela and SL2 *Drosophila* cells	Both Sp1 and Sp3 associate with HDAC1 and p300P300 and CBP (braun 2001)	TSA is a HDAC inhibitor and therefore would be expected to increase Sp1/3 actetylation, however, this is not shown directlySp3 inhibitory domain lysine is acetylated and acts as a repressor, Sp3 purified from transfected insect cells lacks this acetylation and acts as a transcriptional activator	EMSA using Sp1 consensus sequence; ChIP	No change in Sp1/Sp3 binding	RII promoter luciferase reporter construct transfected into cells	TSA treatment enhanced activity	[61]
-	MCF-7 (T5) cell lysate	HDAC1 and HDAC2 are associated with Sp1 and Sp3HDAC2 (davie 2003 Nutr prot in cancer prevention)	-	-	-	-	-	[62]
TSA	MIA PaCa-2 pancreatic cancer cells	Sp1 forms a multiprotein complex with NF-Y, P300, PCAF and HDAC1	TSA treatment enhanced the acetylation of Sp1	-	-	TβRII promoter luciferase construct	Activation	[28]
TAS-103 treatment	Human epidermoid cancer KB cells; Human glioblastoma T98G cells; MCF-7 breast cancer cells	P300	Acetylation of Sp1	-	-	SV40 promoter	Activation	[30]
Phorbol 12-myristate 13-acetate (PMA)	Human epidermoid carcinoma A431 cells	HDAC1; p300	Sp1 is acetylated at K703 and is deacetylated upon PMA treatment	-	-	12(S)-lipoxygenase promoter -luciferase reporter construct	Mutant K703A Sp1 (deacetylated) showed reduced activation capacity	[26]
Butyrate treatment	Caco-2 cells	P300	Sp3 acetylation	GC box from the hIGFBP-3 promoter	Increased binding of acetylated Sp3	hIGFBP-3 mRNA levels	Repression	[31]
Butyrate treatment	HCT116 cells	-	Acetylation of Sp1 reduces binding, increased Sp3 binding	EMSA	-	BAK	Activation	[36]
**Glycosylation**								
**Treatment**	**Cell/Tissue**	**Interacting Proteins**	**PTM Including Residues/Location**	**DNA Binding**		**Transcription Effects**		**Ref.**
				**Probe/Assay**	**Effect**	**Promoter**	**Activation/Repression**	
Wheatgerm agglutinin (WGA) binding of glyscoylated Sp1	Hela cell nuclear extracts	-	Glycosylation at Sp1 Serine/Threonine residues	DNAse I protein experiments	No effect on DNA binding	SV40	Decreased transcription 3-4 fold	[37]
Under glucose starvation, cAMP stimulation with forskolin treatment, results in nearly complete deglycosylation of Sp1.	NRK cells	-	Sp1 deglycosylation, leading to proteosome targeting	EMSA using an Sp1 consensus sequence	Virtual loss of DNA binding activity	-	-	[63]
Mutation of a glycosylation site in a fragment of Sp1	Hela	-	Mutation of the glycosylation site should cause deglycosylation of the Sp1 fragment	-	-	Gal4 dependant luciferase reporter construct	Activation with both mutant and wild type Sp1 fragments. However in an in vitro assay only the glycosylated form could bind to TAF-110	[43]
Glycolysis inhibition by 2-DG (non metabolizable glucose analogue)	Hela	-	-	HPV18 URR Sp1 binding sequence	No effect on DNA binding	Luciferase reporter assay	Repression	[39]
Glutamine or glucosamine treatment	Caco-2	-	Increased O-glycosylation of Sp1 leading to its translocation into nucleus	GC boxes of the ASS promoter used as a probe for EMSA	Increased binding	-	-	[40]
Insulin treatment	H-411E rat hepatoma cell lineDrosophila SL2 cells used for reporter assay	-	Increased total and O-GlcNAc-modified Sp1 primarily in the nucleus and induced CaM I gene transcription	-	-	Cotransfection of Sp1 and rat CaM I promoter containing Sp1 sites in SL2 cells	Activation	[41, 64]
Glucose deprivation or treatment of cells with 6-diazo-5-oxo-L-norleucine	NB4 cells	-	Deglycosylation of Sp1	ChIP for hTERT promoter	No effect	qRT-PCR for hTERT gene	No effect on transcription	[44]
Insulin treatment	H-411E rat hepatoma cell line	-	Glycosylation followed by phosphorylation at Serines 613, 642, 699, 703 and threonine 641.	-	-	CaM I mRNA	Levels of CaM I mRNA increased steadily with time following insulin exposure	[42]
High glucose	Glomerular mesangial cells	-	Glycosylation of Sp1	PAI-1 promoter used in EMSA	No effect on DNA binding	PAI-1 promoter	Activation	[38]
**Sumoylation**								
**Sp-Protein**	**Cell/Tissue**	**Interacting Proteins**	**PTM Including Residues/Location**	**DNA Binding**		**Transcription Effects**		**Ref.**
				**Probe/Assay**	**Effect**	**Promoter**	**Activation/Repression**	
Sp3	MCF-7E		Sp3 and it’s shorter isoforms (M1 and M2) are sumolyated at K551			PSA promoter	K551R substitution led to a marginal increase of transactivation for full length Sp1. The same substitution in the M1 isoform markedly enhanced transactivation	[46]
Sp1	MCF-7E		Sp1 is sumolated at K16, governing processing	-	-	Synthetic, p21	Sumolation of Sp1 is repressive of transcription	[45]
Sp3	SW480			-	-	SRC-1A promoter	Differential according to isoform	[47]
